# Impact of body weight change on clinical outcomes in patients with idiopathic pulmonary fibrosis receiving pirfenidone

**DOI:** 10.1038/s41598-022-22449-w

**Published:** 2022-10-17

**Authors:** Tae Hun Kim, Yune-Young Shin, Hyung-Jun Kim, Myung Jin Song, Yeon Wook Kim, Sung Yoon Lim, Yeon Joo Lee, Jong Sun Park, Young-Jae Cho, Jae Ho Lee, Choon-Taek Lee, Byoung Soo Kwon

**Affiliations:** 1 Division of Pulmonary and Critical Care Medicine, Department of Internal Medicine, Seoul National University Bundang Hospital, 82 Gumi-Ro 173 Beon-Gil, Bundang-Gu, Seongnam, Gyeonggi-Do 13620 South Korea; 2 Division of Pulmonary and Critical Care Medicine, Department of Internal Medicine, Dongsan Hospital, Keimyung University School of Medicine, Daegu, South Korea; 3 Division of Pulmonary and Critical Care Medicine, Department of Internal Medicine, Ewha Womans University, Seoul, South Korea; 4 Department of Internal Medicine, Seoul National University College of Medicine, Seoul, South Korea

**Keywords:** Medical research, Risk factors

## Abstract

There have been limited studies on the association between prognosis and body weight change in patients with idiopathic pulmonary fibrosis (IPF). This single-center retrospective observational study evaluated the impact of weight loss on outcomes in Korean patients with IPF receiving pirfenidone at a tertiary medical institution. We analyzed 215 IPF patients prescribed pirfenidone from January 1st, 2015 to December 31st, 2019. The patients were categorized into maintained weight (MW; weight gain or loss < 5%/year) and reduced weight (RW; weight loss ≥ 5%/year) groups. The mean age was 71.8 years and 175 (81.4%) were male. There were 54 (25.1%) patients in the RW group. All patients showed a decrease in body weight (baseline vs. after 1 year; 64.1 kg vs. 62.8 kg, *P* < 0.001). Although baseline lung function showed a difference, there was no difference in the rate of change (forced vital capacity [% of predicted]; *P* = 0.221, diffusing capacity of the lung for carbon monoxide [% of predicted]; *P* = 0.973). The MW group had a lower risk of all-cause mortality (*P* < 0.001). Weight loss appeared to be a significant risk factor for mortality in patients with IPF. Not only disease control with antifibrotic agents, but also efforts to prevent weight loss may be necessary.

## Introduction

Idiopathic pulmonary fibrosis (IPF) is an interstitial lung disease characterized by progressive loss of lung function^1^. Although the prognosis of IPF is generally poor and the condition is often fatal, the course of progression is variable, with the median survival in untreated patients being approximately 3 years from diagnosis^2^. The identification of prognostic factors for disease progression is, therefore, an important and active area of interest^3^. Comorbidities, old age, smoking, decline in forced vital capacity (FVC), GAP score, and exercise capacity are established predictors of mortality in patients with IPF^4,5^.

Body weight and body mass index (BMI) could be easily measured in clinical practice, and previous studies have suggested that lower BMI^6,7^ or weight loss^8^ may be associated with a worse prognosis in patients with IPF. A recent study^9^ analyzing the degree of weight loss and prognosis according to BMI in fibrotic interstitial lung disease (ILD) including IPF also suggests that weight and BMI reduction are independent risk factors for 1-year mortality. However, studies of nintedanib, pirfenidone^10^, and those mentioned above^6–8^ have analyzed prognoses related to acute exacerbations and deterioration of lung function. In addition, a previous study^11^ reported the prevalence of malnutrition based on the fat-free mass index as 28%. In another recent study^12^, malnutrition and decreased food intake were associated with poor IPF outcomes. However, real-world data on long-term weight loss and clinical outcomes in Asians are still lacking. Thus, it remains unclear whether weight changes are associated with prognosis and changes in pulmonary function tests (PFTs) in patients with IPF being treated with pirfenidone.

We hypothesized that changes in body weight during treatment of IPF could be related to the prognosis of IPF and changes in PFTs, including FVC or diffusing capacity for carbon monoxide (DL_CO_). In our study, we assessed body weight change during treatment with pirfenidone and analyzed the associations between changes in body weight and clinical outcomes in Korean patients with IPF.

## Results

### Baseline characteristics

**Figure 1:**
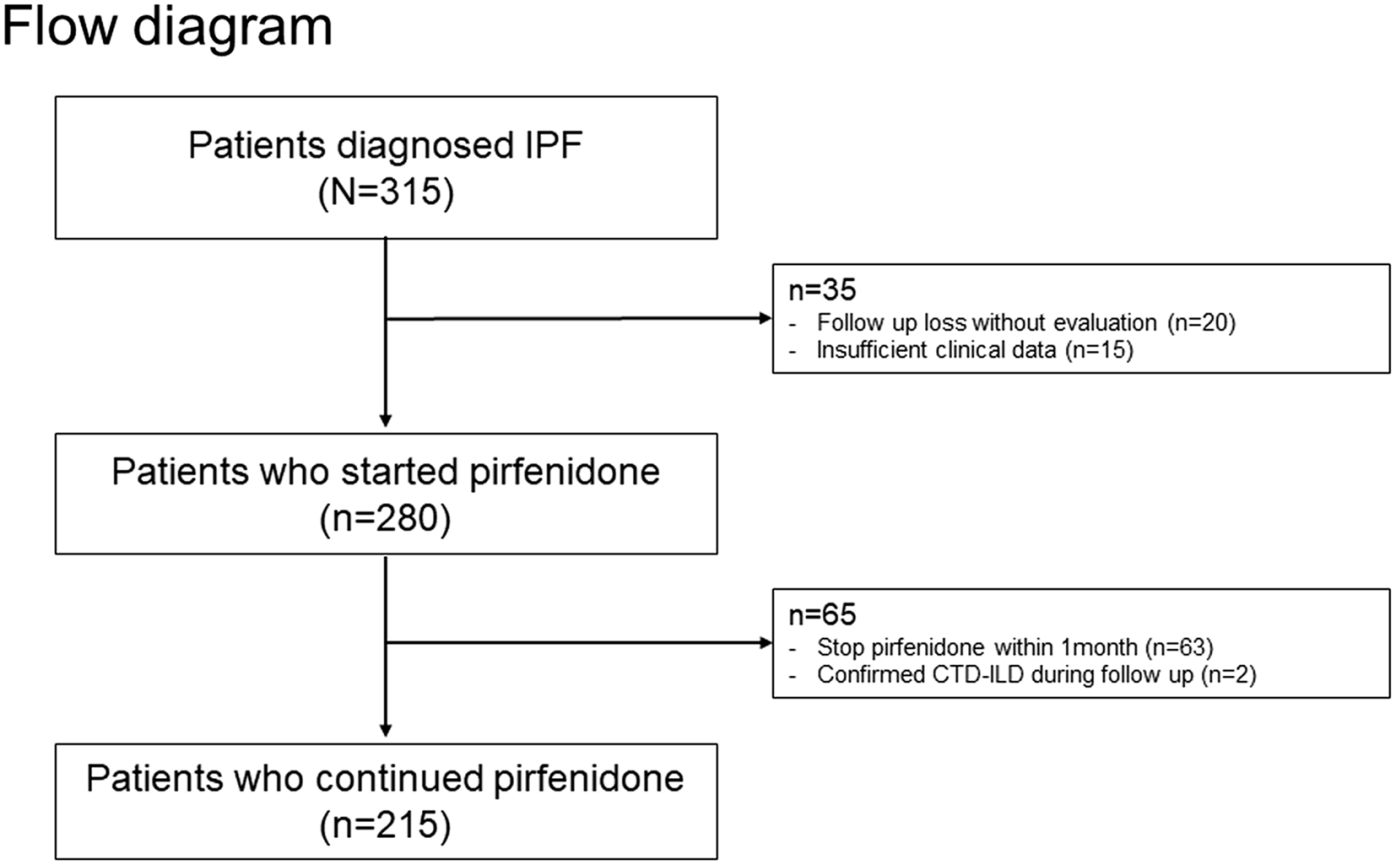
Flow diagram of the study population. *IPF* idiopathic pulmonary fibrosis, *CTD* connective tissue disease, *ILD* interstitial lung disease.

In total, 215 patients were included in the study population (Fig. 1). The mean (± standard deviation [SD]) age of the patients was 71.8 (± 7.4) years, and 175 (81.4%) patients were male. The baseline BMI of patients was 24.0 (± 2.9) kg/m^2^, and 28 (13.0%) patients were diagnosed with IPF by surgical lung biopsy. There were 169 (78.6%) smokers in the study population. The mean (± SD) ages of the maintained weight (MW) and reduced weight (RW) groups were 71.2 (± 7.2) and 73.8 (± 7.7) years, respectively, with a statistically significant difference (*P* = 0.032). The proportion of males was higher in the MW group than in the RW group: 134 (83.2%) and 41 (75.9%), respectively, but this difference did not have statistical significance (*P* = 0.233). In addition, the RW group was more likely to have lower BMI, baseline body weight, and lung function (Table 1).

**Table 1:** Baseline demographics of the patients.

	Maintained weight* (n = 161, 74.9%)	Reduced weight** (n = 54, 25.1%)	*P*-value
Age (years)	71.2 ± 7.2	73.8 ± 7.7	0.032
**Sex**			
Male	134 (83.2%)	41 (75.9%)	0.233
Female	27 (16.8%)	13 (24.1%)	
BMI (kg/m^2^)	24.4 ± 2.9	23.0 ± 2.9	0.002
Baseline body weight (kg)	65.3 ± 9.3	60.1 ± 9.0	< 0.001
**Smoking history**			
Never smoker	35 (21.7%)	11 (20.4%)	0.692
Ever smoker	126 (78.3%)	43 (79.6%)	
**Comorbidities**			
Hypertension	48 (29.8%)	12 (22.2%)	0.282
Diabetes mellitus	41 (25.5%)	12 (22.2%)	0.635
COPD	21 (13.0%)	2 (3.7%)	0.055
Malignancy	23 (14.3%)	3 (5.6%)	0.089
**Pulmonary function test**			
FVC (% of predicted)	81.1 ± 15.5	71.1 ± 16.8	< 0.001
DL_CO_ (% of predicted)	72.0 ± 20.5	62.3 ± 20.7	0.004
**Pirfenidone treatment**			
Dose adjustment	56 (34.8%)	19 (35.2%)	0.957

Data are expressed as mean ± standard deviation or counts (%).

*BMI* body mass index, *FVC* forced vital capacity, *DL*_*CO*_ diffusing capacity of the lung for carbon monoxide, *COPD* chronic obstructive pulmonary disease.

*Maintained weight group is weight gain or weight loss < 5%/year, **Reduced weight group is weight loss ≥ 5%/year.

Patients showed a statistically significant decrease in body weight and BMI during the follow-up (baseline vs. after 1 year [kg] 64.1 vs. 62.8, *P* < 0.001; BMI (kg/m^2^) 24.1 vs. 23.7, *P* < 0.001; Table S.1). There were 161 (74.9%) patients in the MW group and 54 (25.1%) patients in the RW group.

A total of 75 patients required pirfenidone dose adjustment (56 in the MW group vs. 19 in the RW group). Among those who had a history of pirfenidone dose reduction, gastrointestinal side effects were the most common side effect in both the MW and RW groups (n = 17 [10.6%] and n = 14 [25.9%], respectively; Table S.2). However, there was no statistical relationship between weight loss and a dose reduction history of pirfenidone (*P* = 0.957; Table S.3).

### Lung function decline

**Figure 2:**
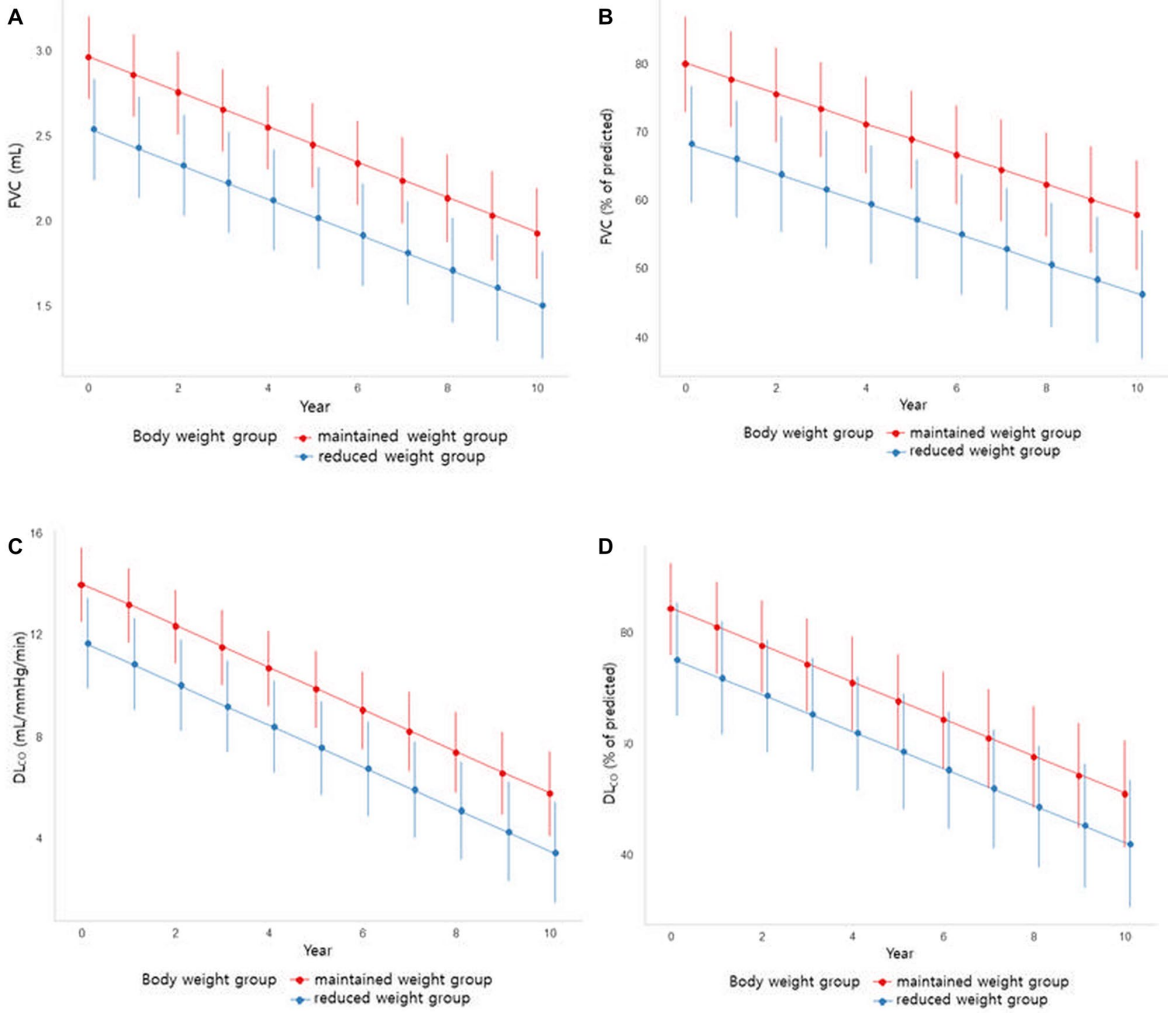
Linear mixed-effect model of pulmonary function trends. (**A**) Predicted mean FVC (mL) of body weight group with 95% CI. (**B**) Predicted mean FVC (% of predicted) of body weight group with 95% CI. (**C**) Predicted mean DLCO (mL/mmHg/min) of body weight group with 95% CI. (**D**) Predicted mean DLCO (% of predicted) of body weight group with 95% CI. *P*_Interaction_ analysis of interaction (type III) according to the two groups of body weight change (maintained weight vs. reduced weight) showed there was no interaction with time for each lung function change result. FVC (mL) *P*_interaction_ = 0.214, FVC (% of predicted) *P*_interaction_ = 0.221, DL_CO_ (mL/mmHg/min) *P*_interaction_ = 0.429, DL_CO_ (% of predicted) *P*_interaction_ = 0.973.

The mean annual changes in lung function were as follows: FVC (% of predicted), − 2.2%/year (95% confidence interval [CI] − 2.7, − 1.6); and DL_CO_ (% of predicted), − 3.9%/year (95% CI − 4.5, − 3.2) (Table 2). When the interaction effects according to the two groups of body weight change (MW vs. RW) were analyzed, there was no difference in the rate of change (forced vital capacity [% of predicted]; *P*_interaction_ = 0.221, diffusing capacity of the lung for carbon monoxide [% of predicted]; *P*_interaction_ = 0.973] (Fig. 2).

**Table 2:** Average annual rate of changes in lung function.

	Average annual rate of change	95% CI
FVC (mL/year)	− 102	− 116, − 87
FVC (% of predicted/year)	− 2.2	− 2.7, − 1.6
DL_CO_ (mL/mmHg/min/year)	− 0.9	− 1.0, − 0.8
DL_CO_ (% of predicted/year)	− 3.9	− 4.5, − 3.2

*CI* confidence interval, *FVC* forced vital capacity, *DL*_*CO*_ diffusing capacity of the lung for carbon monoxide.

There was a statistically significant difference in lung function between the maintained and reduced weight groups. The estimated differences in lung function were as follows: FVC (% of predicted), − 11.7%, *P* < 0.001; DL_CO_ (% of predicted), − 9.1%, *P* = 0.004 (Table 3, Fig. 2).

**Table 3:** Linear mixed effect model of pulmonary function tests corrected for sex, age and smoking status.

	Maintained vs. reduced weight group		
	Estimate	95% CI	*P*-value
FVC (mL)	− 425	− 600, − 250	< 0.001
FVC (% of predicted)	− 11.7	− 19.7, − 6.6	< 0.001
DL_CO_ (mL/mmHg/min)	− 2.3	− 3.4, − 1.3	< 0.001
DL_CO_ (% of predicted)	− 9.1	− 15.2, − 3.0	0.004

*CI* confidence interval, *FVC* forced vital capacity, *DL*_*CO*_ diffusing capacity of the lung for carbon monoxide.

### Survival Outcomes

**Figure 3:**
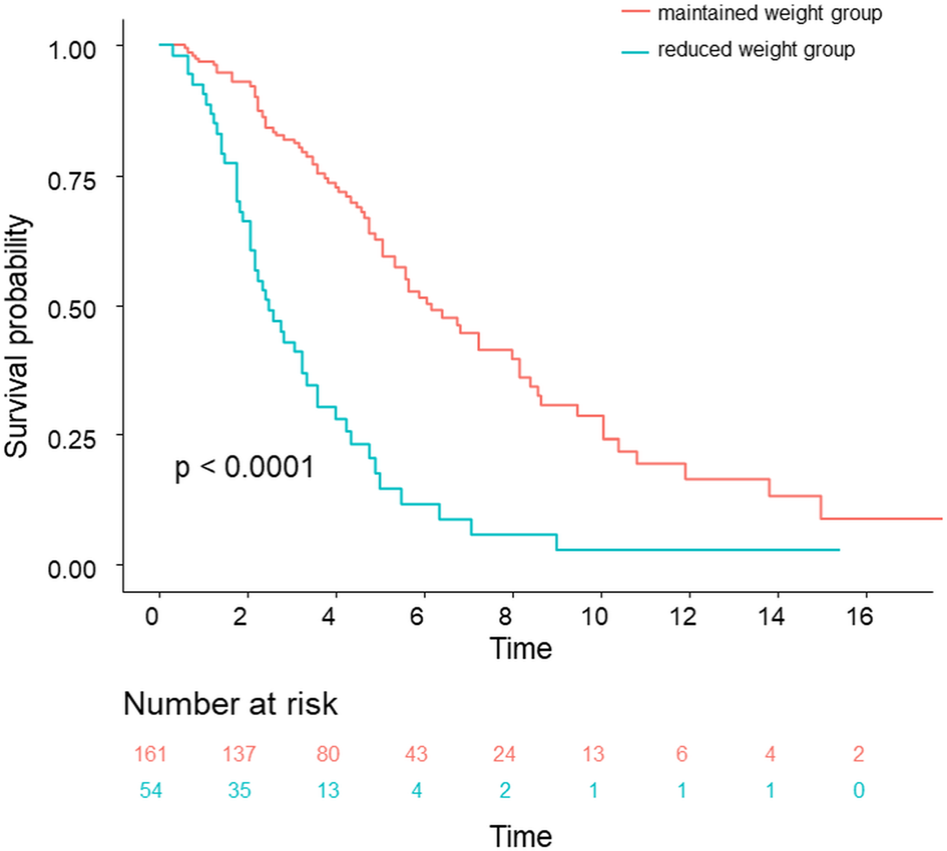
Time-to-event analysis of all-cause mortality, stratified by an annualized percentage change in body weight categories. Maintained weight group, preserved body or < 5%/year weight loss; Reduced weight group, ≥ 5%/year weight loss.

In time-to-event analysis, patients in the MW group had a significantly lower risk of experiencing all-cause mortality than patients who experienced a > 5%/year loss (*P* < 0.001) (Fig. 3). In univariate Cox proportional-hazards analysis, body weight loss was an independent risk factor for all-cause mortality (hazard ratio [HR] 3.270, 95% CI 2.254, 4.743, *P* < 0.001) along with age as well as lower baseline FVC and DL_CO_. In multivariate Cox proportional-hazards analysis, weight loss remained a significant factor (HR 2.358, 95% CI 1.572, 3.537, *P* < 0.001) along with age and lower baseline DL_CO_ (Table 4). Weight loss was also analyzed as a risk factor for mortality in the time-dependent Cox regression analysis for the time point of 5% weight loss (HR 1.751, 95% CI 1.229, 2.494, *P* = 0.002) (Table S.4).

**Table 4:** All-cause mortality from time since IPF diagnosis, for the significant baseline predictors at diagnosis as estimated by the Cox proportional-hazards ratio model.

Characteristic	Univariable analysis			Multivariable analysis		
	HR	95% CI	*P*-value	HR	95% CI	*P*-value
Male sex	1.097	0.692–1.739	0.6949	–	–	–
Age	1.057	1.030–1.085	< 0.001	1.054	1.029–1.081	< 0.001
**Smoking**						
Ever smoking	0.820	0.543–1.237	0.3441	–	–	–
Baseline BMI (kg/m^2^)	0.958	0.902–1.018	0.1694	–	–	–
Reduced body weight	3.270	2.254–4.743	< 0.001	2.358	1.572–3.537	< 0.001
**Baseline PFT**						
FVC (% of predicted)	0.976	0.965–0.988	< 0.001	0.992	0.978–1.006	0.2682
DL_CO_ (% of predicted)	0.974	0.965–0.983	< 0.001	0.980	0.968–0.991	< 0.001

*BMI* body mass index, *FVC* forced vital capacity, *DL*_*CO*_ diffusing capacity of the lung for carbon monoxide, *HR* Hazard Ratio, *CI* confidence Interval.

## Discussion

In this study, significant body weight loss was observed in approximately 25% of patients treated with pirfenidone. Pirfenidone has many side effects and is frequently associated with gastrointestinal symptoms like nausea, anorexia, diarrhea, liver function test abnormalities, or cutaneous side effects^13^. During follow-up, IPF patients prescribed pirfenidone showed a tendency to lose weight and have decreased BMI during follow-up periods, but there was no statistically significant relationship between body weight loss and pirfenidone dose adjustment due to side effects and compliance. Moreover, body weight loss of ≥ 5% in patients with and without pirfenidone treatment (25.1% vs. 7.8%; data not shown) exhibited similar results as in the previous literature^14^. Although it was found that weight loss was not related to the rate of deterioration in lung function, patients experiencing significant weight loss during treatment had a higher risk of all-cause mortality compared to patients who maintained their weight. As in previous studies, age and baseline DL_CO_ (% of predicted) were identified as risk factors for poor outcomes; in this study, significant weight loss was also confirmed to be a significant prognostic factor.

The results of this study are similar to the results of other studies that assessed the relationship between BMI, weight loss, and clinical outcomes of IPF. The studies reported that weight loss and BMI are associated with an increased risk of mortality in IPF patients, which is regarded as a marker of disease progression^8,14–16^. A study in patients with a placebo arm reported the proportion of patients with a decline in FVC by more than 10% or death up to 1 year was higher in patients with an annualized weight loss ≥ 5% compared to the patients not experiencing ≥ 5% weight loss^10^. The results from our analysis and the results from previous studies suggest that weight loss during pirfenidone treatment may be associated with worse clinical outcomes, including a decline in PFTs, in patients with IPF^8,14–16^.

In a study of IPF patients treated with nintedanib, the converse results were reported for weight loss during a 52-week period; they reported a greater rate of decline in lung function in patients who maintained weight compared to those who experienced reduced body weight by over 5%^14^. In the study, the mean BMIs in the maintained weight and weight loss groups were 27.9 kg/m^2^ and 28.4 kg/m^2^, respectively, both meeting the criteria for ‘overweight’ according to the world health organization (WHO) classification^17^. Changes in lung function due to weight loss may show different trends depending on the initial degree of obesity or race. The nintedanib study was different from ours in that the majority of patients were white and the BMI of the patients in our study was normal. There were many intersections during the follow-up period; an observational study on long-term changes after 52 weeks is necessary for nintedanib as well. Given the lack of research about the risk factors for IPF patients treated with pirfenidone, this study is meaningful as it provides real-world data analyzing the long-term outcomes from over 5 years.

This study had several limitations. First, it was a retrospective observational study from a single center; prospective datasets are required to evaluate whether weight changes are independently associated with clinical outcomes. Nevertheless, this study showed consistent results in terms of weight change and prognosis. Second, we included patients who received a follow-up PFT, which means patients who were unable to receive a follow-up PFT due to death or rapid worsening of IPF were excluded. We may thus have focused on patients with a better condition and prognosis. However, the median survival of all enrolled patients in this study was 60 months, and considering that the median survival of patients using pirfenidone in another study was 6.87 years^18^, it cannot be said that the initial condition of our patients was good. Third, we could not obtain information on nutrition status or socioeconomic status, and intensity of daily activity. Fourth, we could not obtain enough data to evaluate cardiac function; heart failure may affect sodium retention and thus may have a potential effect on weight. Fifth, pirfenidone can result in weight loss in patients with IPF^19^, but we could not evaluate an association between the cumulative dose of pirfenidone and intensity of weight loss. Sixth, in South Korea, nintedanib for the treatment of IPF is not covered by insurance yet, so we could not make a comparison with the nintedanib group. Finally, looking at the baseline characteristics of patients with significant weight loss, although baseline BMI was not analyzed as a significant factor in survival in the study, compared to the patients who maintained their weight, patients with significant weight loss had lower baseline BMI and body weight; in addition, they tended to have poorer baseline lung function and tended to be older. Given the baseline characteristics of the reduced weight group, there is a limitation relating to whether significant weight loss was a result of disease progression or a treatable trait.

In this study, 25.1% of patients experienced an average annual weight loss of 5% or more, and they had poor lung function and prognosis. In daily practice, body weight is noninvasively measured and is a relatively easy, measurable indicator of prognosis in IPF patients. For this reason, it may be necessary not only to control the disease with antifibrotic agents but also to provide efforts to prevent weight loss and maintain physical status with nutritional support and the maintenance of muscle mass with rehabilitation^20^. The nutritional status of patients with IPF requires further study, including the impact of nutrition support teams or pulmonary rehabilitation. Further well-designed prospective studies on the traits requiring treatment are needed.

## Conclusions

In this study, approximately 25% of the IPF patients on pirfenidone treatment experienced significant weight loss of 5% or more per year, and these patients had a poor prognosis. Although well-designed studies of treatable factors are needed to improve the outcomes of patients with IPF and continue treatment, it may be helpful to evaluate weight trends during follow-up, as well as adjust the dose of antifibrotic agents and track lung function. In addition, efforts to prevent weight loss may also be necessary.

## Methods

### Study design and participants

This study was a single-center retrospective observational study conducted in a tertiary referral hospital in South Korea from January 1st, 2015 to December 31st, 2019. The diagnosis of IPF met the Society consensus definition of the official American Thoracic Society/European Respiratory Society/Japanese Respiratory Society/and Latin American Thoracic Society statement^1^. In total, 315 patients were identified as meeting the following criteria: (1) a diagnosis of IPF on the basis of high-resolution computed tomography (HRCT) and lung biopsy if available, and (2) being prescribed pirfenidone during follow-up. We excluded patients who: (1) were lost to follow-up without additional PFT data, (2) were transferred to other centers resulting in insufficient clinical information, (3) received pirfenidone for less than 1 month, and (4) had a confirmed connective tissue disease related to ILD during follow-up while receiving pirfenidone. The HRCT imaging was reviewed by an experienced chest radiologist. Among the above IPF patients, we reviewed 215 patients after applying the above exclusion criteria.

### Data collection

Clinical data were collected from the patients’ electronic medical records. The following data were recorded: (1) demographics and comorbidities, (2) dose of prescribed pirfenidone (3) patients’ height and body weight obtained from the serial PFT, and (4) survival records obtained from the Ministry of the Interior and Safety. Each time a PFT was performed, the body weight and height were measured and recorded in the laboratory, and this was used for analysis. The annualized percentage change in body weight was categorized based on the US Food and Drug Administration (FDA) guidance for developing products for weight management, which recommends an annualized weight loss of ≥ 5% loss of reference value for weight loss^17^.

The ratio of weight change was calculated by dividing the difference between the latest and baseline weight (body weight at the time of first PFT performed at diagnosis) by baseline body weight. The annualized percentage of weight change was calculated by multiplying the ratio of weight change by 100 and dividing by the periods (12 months) [(*latest body weight*-*baseline body weight*)×100/*baseline body weight*/12 months]. We categorized the annualized percentage of weight change in body weight into two groups: MW group (weight gain or weight loss < 5%/year during follow-up periods) and RW group (weight loss ≥ 5%/year during follow-up periods). Annual changes in lung function were also calculated as changes over time in the PFT results. We performed followed up PFT every 6–12 months from the baseline study.

### Statistical analysis

Baseline demographics and characteristics were reported descriptively. A paired *t* test was used to compare changes in body weight and BMI during follow-up. The correlation between weight loss and dose reduction events of pirfenidone due to side effects was evaluated using chi-square testing. Student’s *t* test was used for the descriptive analysis comparisons. We used a linear mixed-effect model to analyze trends of indices of pulmonary function tests such as FVC and DL_CO_. Time-to-event analyses of survival are presented according to the presence or absence of significant weight loss. The log-rank test was used to evaluate the Kaplan–Meier curve for survival. The Cox proportional hazards test was used for the analysis of mortality. Variables with a P-value < 0.2 in the univariate analysis were entered into the multivariable analysis and selected by the backward log-likelihood ratio method. Since the weight change is a time-dependent variable, the time-dependent Cox proportional hazard model was used for the analysis considering the time of weight loss of 5% or more. Variables that did not satisfy the proportional hazards assumption were categorized, and stratification was applied even after the categorization did not meet the assumption. In this case, although P-value < 0.05 in univariable analysis, hazard ratio results did not appear in multivariable analysis. A P-value of < 0.05 was considered statistically significant. Statistical analyses were performed using IBM® SPSS® Statistics version 25 (IBM Corp., Armonk, NY, USA) and R version 4.1.1.

### Ethics approval and consent to participate

The study protocol was approved by the Institutional Review Board (IRB) of Seoul National University Bundang Hospital (IRB No. B-2002-592-103), and is consistent with the principles of the Declaration of Helsinki. The requirement for informed consent was waived by IRB of Seoul National University Bundang Hospital because of the retrospective nature of the study.

## Supplementary Information

Supplementary Tables.

## Data Availability

The data that support the findings of this study are not openly available due to the fact that consent to share data was not obtained from participants. However, the datasets used and analyzed in the current study are available from the corresponding author on reasonable request.
