# Supplementary material for: Impact of body weight change on clinical outcomes in patients with idiopathic pulmonary fibrosis receiving pirfenidone
Source: Sci Rep. 2022 Oct 17;12:17397. doi: 10.1038/s41598-022-22449-w (PMC9576723; doi:10.1038/s41598-022-22449-w)
Supplement: Supplementary file 1 — Supplementary Tables. [file 41598_2022_22449_MOESM1_ESM.docx]

**Impact of body weight change on clinical outcomes in patients with idiopathic pulmonary fibrosis receiving pirfenidone**

**Running head: Body weight in IPF patients receiving pirfenidone**

Tae Hun Kim^1,2^, Yune-Young Shin^3^, Hyung-Jun Kim^1^, Myung Jin Song^1^, Yeon Wook Kim^1^, Sung Yoon Lim^1^, Yeon Joo Lee^1^, Jong Sun Park^1^, Young-Jae Cho^1^, Jae Ho Lee^1^, Choon-Taek Lee^1^, Byoung Soo Kwon^1,^*

^1^Divisions of Pulmonary and Critical Care Medicine, Department of Internal medicine, Seoul National University College of Medicine, Seoul National University Bundang Hospital, Seongnam, South Korea

^2^Divisions of Pulmonary and Critical Care Medicine, Department of Internal Medicine, Dongsan Hospital, Keimyung University School of Medicine, Daegu, South Korea

^3^Divisions of Pulmonary and Critical Care Medicine, Department of Internal Medicine, Ewha Womans University, Seoul, South Korea

***Corresponding author:** Byoung Soo Kwon, MD^1^*

^1^Divisions of Pulmonary and Critical Care Medicine, Department of Internal medicine, Seoul National University College of Medicine, Seoul National University Bundang Hospital, 82 Gumi-ro 173 Beon-gil, Bundang-gu, Gyeonggi-do 13620, Korea

Tel: +82-31-787-7026, Fax: +82-31-787-2204

E-mail: [bskwon82@gmail.com](mailto:bskwon82@gmail.com)

**Supplementary data**

**Table S.1** Changes in the Body weight and Body Mass Index over one year

| Body weight (kg, ± SD) | | *P*-value |
| --- | --- | --- |
| Baseline | After one year | <0.001 |
| 64.1 ±9.6 | 62.8 ±10.1 |  |
| Body mass index (BMI; kg/m^2^, ±SD) | | *P*-value |
| Baseline | After one year | <0.001 |
| 24.1 ±3.0 | 23.7 ±3.2 |  |

**Table S.2** Side effects of pirfenidone

| Pirfenidone | Annualized percent change in body weight (All, N=215) | | *P*-value |
| --- | --- | --- | --- |
|  | Maintained weight  (n=161, 74.88%) | Reduced weight  (n=54, 25.11%) |  |
| Dose reduction | 56 (34.8%) | 19 (35.2%) | 0.957 |
| Adverse events | GI 17 (10.6%)  Skin 10 (6.2%)  Weakness 3 (1.9%)  LFT abnormality 5 (3.1%) | GI 14 (25.9%)  Skin 1 (1.9%)  Weakness 1 (1.9%)  LFT abnormality 1 (1.9%) |  |

GI, gastrointestinal; LFT, liver function test

**Table S.3** Association between pirfenidone dose reduction and weight loss

|  | | Pirfenidone dose reduction history | | Total |
| --- | --- | --- | --- | --- |
|  |  | none | present |  |
| Group | Maintained weight | 105 (65.2%) | 56 (34.8%) | 161 (100%) |
|  | Reduced weight | 35 (64.8%) | 19 (35.2%) | 54 (100%) |

Chi-square test; *P*=0.957

**Table S.4** Time-dependent Cox regression model of mortality and weight loss

| Variables | | Univariable | | | Multivariable | | |
| --- | --- | --- | --- | --- | --- | --- | --- |
|  |  | HR | 95% CI | *P*-value | HR | 95% CI | *P*-value |
| Female |  | 1.141 | 0.714 – 1.824 | 0.582 |  |  |  |
| Age ≥70 |  | 2.108 | 1.401 – 3.171 | <0.001 | 1.963 | 1.297 – 2.970 | 0.001 |
| Ever smoker |  | 0.977 | 0.650 – 1.468 | 0.910 |  |  |  |
| Hypertension |  | 0.821 | 0.536 – 1.257 | 0.365 |  |  |  |
| Diabetes mellitus |  | 0.902 | 0.602 – 1.351 | 0.616 |  |  |  |
| COPD |  | 0.407 | 0.232 – 0.715 | 0.002 | 0.415 | 0.230 – 0.748 | 0.003 |
| Malignancy |  | 0.881 | 0.534 – 1.452 | 0.618 |  |  |  |
| Body mass index |  | 0.930 | 0.875 – 0.988 | 0.019 | 0.954 | 0.898 – 1.014 | 0.012 |
| Weight loss ≥5% |  | 2.864 | 2.061 – 3.980 | <0.001 | 1.751 | 1.229 – 2.494 | 0.002 |
| FVC (%) 50-79 | Ref: FVC (%)≥80 | 2.272 | 1.582 – 3.263 | <0.001 |  |  |  |
| FVC (%) 30-49 | Ref: FVC (%)≥80 | 2.267 | 0.807 – 6.373 | 0.121 |  |  |  |
| DL_CO_ (%) 50-79 | Ref: DL_CO_ (%)≥80 | 2.170 | 1.441 – 3.267 | <0.001 | 1.681 | 1.119 – 2.523 | 0.012 |
| DL_CO_ (%) 30-49 | Ref: DL_CO_ (%)≥80 | 3.342 | 1.889 – 5.913 | <0.001 | 2.878 | 1.668 – 4.968 | <0.001 |
| DL_CO_ (%) <30 | Ref: DL_CO_ (%)≥80 | 15.839 | 6.722 – 37.323 | <0.001 | 8.661 | 4.646 – 16.147 | <0.001 |

COPD, chronic obstructive lung disease; FVC, forced vital capacity; DL_CO_, diffusing capacity for carbon monoxide; HR, Hazard Ratio; CI, confidence Interval
